# Associations between physical fitness components and metabolic syndrome in middle-aged adults: a cross-sectional study using relative strength indicators and ROC analysis

**DOI:** 10.3389/fpubh.2025.1712376

**Published:** 2025-11-17

**Authors:** Zhanfei Zheng, Haoliang Qing, Lingfeng Zhang, Changyuan Duan

**Affiliations:** 1 Sports Coaching College, Beijing Sport University, Beijing, China; 2 Department of Sports Science, Wenzhou Medical University, Wenzhou, Zhejiang, China; 3 School of Physical Education and Health, Wenzhou University, Wenzhou, Zhejiang, China; 4 Department of Physical Education, Sichuan Electronic and Mechanic Vocational College, Mianyang, Sichuan, China; 5 Department of Physical Education, Gangneung-Wonju National University, Gangneung, Republic of Korea

**Keywords:** metabolic syndrome, middle-aged adults, relative strength, isokinetic strength, grip strength, cardiorespiratory fitness, ROC curve

## Abstract

**Introduction:**

Metabolic syndrome (MetS) is a major risk factor for cardiovascular disease (CVD) and type 2 diabetes, particularly in middle-aged populations. Physical fitness, especially muscular strength and cardiorespiratory capacity, has emerged as a pragmatic indicator of metabolic health. In this cross-sectional study, we investigated the association between multiple fitness components and the presence of MetS in middle-aged adults, with emphasis on weight-normalized indices.

**Methods:**

A total of 570 middle-aged adults (369 male, 201 female) were enrolled in this cross-sectional study. Assessments included body composition measured by bioelectrical impedance analysis (appendicular skeletal muscle mass, ASM), handgrip strength, isokinetic lower-limb strength at 60°/s using the HUMAC NORM system, and cardiorespiratory fitness (CRF) assessed by maximal treadmill testing (VO₂max, mL·kg^−1^·min^−1^, Bruce protocol). MetS was defined according to the International Diabetes Federation criteria. Within each sex, logistic regression models [scaled per 1 SD decrease in each exposure; Model 1 adjusted for age; Model 2 adjusted for age and body mass index (BMI)] were used to quantify associations. Receiver operating characteristic analyses with the Youden index were used to identify sex-specific cutoffs.

**Results:**

The participants with MetS showed significantly lower relative grip strength, relative lower-limb strength, and VO₂max than their non-MetS counterparts. A lower ASM ratio was also characteristic of MetS. In age-adjusted models, the decreased ASM ratio and lower relative grip strength were associated with higher odds of MetS, with attenuation after additional adjustment for BMI. ROC analysis yielded pragmatic thresholds for identifying MetS: ASM ratio < 24.0% in male individuals and < 20.0% in female individuals; relative grip strength < 53.3 in male individuals and < 38.0 kg/BW in female individuals; relative lower-limb strength < 5.94 in male individuals and < 5.03 Nm/BW in female individuals; and VO₂max < 24.0 in male individuals and < 19.3 mL·kg^−1^·min^−1^ in female individuals.

**Conclusion:**

Weight-normalized fitness indices, particularly the ASM ratio and relative grip strength, are informative for identifying prevalent MetS in middle-aged adults and support sex-specific screening thresholds suitable for clinical and community practice.

## 1 Introduction

Population aging represents one of the most significant demographic transitions globally, characterized by increasing proportions of older adults within the total population. Current estimates indicate that by 2050, individuals aged 60 and older will comprise approximately 22% of the global population, an increase from 11% in recent decades (1, 2). This demographic shift has substantial implications for public health systems worldwide, primarily through increased prevalence and healthcare burdens associated with age-related chronic diseases, especially cardiovascular diseases (CVDs). Cardiovascular diseases remain among the leading causes of morbidity and mortality globally, responsible for approximately 30% of all deaths, with significantly higher incidence rates among older populations, thereby imposing significant economic and healthcare burdens worldwide (3, 4).

Metabolic syndrome (MetS) is defined as a cluster of metabolic disturbances, including abdominal obesity, elevated blood pressure, dyslipidemia [characterized by elevated triglycerides and reduced high-density lipoprotein cholesterol (HDL-C)], and impaired fasting glucose (FG) (5, 6). MetS significantly increases the risk of developing severe cardiovascular outcomes such as coronary artery disease, stroke, peripheral arterial disease, and type 2 diabetes mellitus (6, 7). Epidemiological studies have demonstrated that individuals diagnosed with MetS exhibit a two- to threefold higher risk of cardiovascular morbidity and mortality compared to healthy counterparts (8, 9). Over recent decades, the global prevalence of MetS has risen dramatically, paralleling accelerating trends in population aging, urbanization, and lifestyle transitions, including increased sedentary behaviors, reduced physical activity, and dietary patterns characterized by excessive caloric intake and unhealthy food choices (10, 11). For example, data from the National Health and Nutrition Examination Survey (NHANES) indicate that approximately one-third of the U.S. adult population meets the criteria for MetS, with even higher prevalence rates reported among older adults (12). Similar rising trends are observed in Asian populations, particularly in China and Korea, reflecting significant increases in abdominal obesity, hypertension, dyslipidemia, and impaired glucose metabolism (11, 13). Therefore, effective strategies for the early identification and prevention of MetS are urgently required to mitigate associated health and economic burdens.

Recent evidence underscores the significance of physical fitness parameters, notably muscle strength and cardiorespiratory fitness (CRF), as critical predictors of metabolic health and cardiovascular outcomes. Skeletal muscle functions as an endocrine organ that releases myokines with systemic effects on glucose and lipid metabolism, providing biological plausibility for linking muscle mass and strength to metabolic risk (14, 15). Grip strength, a widely utilized measure of overall muscular strength, has consistently been associated with the risk of MetS, insulin resistance, and cardiovascular mortality (16, 17). The physiological mechanisms underlying these associations include improved insulin sensitivity, enhanced metabolic rate, and reduced systemic inflammation, mediated by increased muscle mass and strength (15, 18). Similarly, lower-limb strength, assessed using precise isokinetic measurements, has been demonstrated to play a pivotal role in maintaining functional independence and mobility—factors that are crucial for sustaining physical activity levels—and, consequently, reducing MetS risk (13, 19, 20). Furthermore, cardiorespiratory fitness, typically quantified by maximal oxygen uptake (VO₂max), has been established as a robust and independent predictor of metabolic abnormalities and cardiovascular events (21, 22). Reduced cardiorespiratory fitness is associated with compromised cardiovascular and metabolic function, emphasizing the importance of aerobic exercise interventions in preventing MetS and its associated complications. Parallel to our focus on simple strength-related indices, an integrated phenotyping body of literature has emerged that combines adiposity distribution with muscular function to refine cardiometabolic risk assessment beyond body mass index (BMI). Pairing waist circumference (WC) with relative handgrip strength (RGS) improves the identification of metabolic syndrome compared to either measure alone (23). Indices that capture the balance between muscle and adiposity, such as the muscle-to-fat ratio, show consistent associations with cardiometabolic risk in large population datasets (24). Recent proposals also integrate body fat percentage, waist circumference, and grip strength into composite scores for metabolic risk stratification (25). Conceptual models of cardiometabolic-based chronic disease and contemporary reviews of functional body composition similarly advocate moving beyond BMI toward phenotypes that reflect adiposity quality and functional capacity (26, 27). Against this background, our objective was not to derive a new composite index but to establish pragmatic, sex-specific cutoff points for strength-related indices that can serve as building blocks for future integrated models.

Despite the growing recognition of these fitness parameters individually, comprehensive studies simultaneously addressing muscle mass, grip strength, lower-limb strength, and cardiorespiratory fitness in predicting MetS prevalence remain limited. Therefore, this study aimed to quantify sex-specific associations between weight-normalized fitness (RGS, relative lower-limb strength, and VO₂max) and relative muscle mass (appendicular skeletal muscle mass (ASM) ratio) with the prevalence of MetS in middle-aged adults and to derive sex-specific cutoff values using receiver operating characteristic (ROC) analysis.

## 2 Materials and methods

### 2.1 Participants and study design

This cross-sectional study recruited 570 community-dwelling middle-aged adults (369 male and 201 female) from a health examination center in Seoul Special City, South Korea. Participants were excluded if they had a history of cardiovascular or cerebrovascular events (e.g., myocardial infarction and stroke), were undergoing treatment for malignant tumors, or had mobility impairments that prevented physical testing. All participants provided written informed consent, and the study protocol was approved by *the Institutional Ethics Committee of Gangneung-Wonju National University (R2020-1)*.

### 2.2 Data collection and risk factors

#### 2.2.1 Blood pressure measurement

Systolic and diastolic blood pressure (SBP/DBP) were measured in the seated position using an automated oscillometric sphygmomanometer (Omron HEM-907XL, Omron Healthcare Co., Kyoto, Japan) with an appropriately sized cuff. Two readings were obtained 1–2 min apart, and the average was used for analysis. If the two readings differed by >5 mmHg, a third reading was taken, and the mean of the two closest values was recorded (28, 48).

#### 2.2.2 Blood sampling and biochemical assays

After an 8–12 h overnight fast, venous blood was drawn from the antecubital vein. Fasting glucose (FG), triglyceride (TG), and high-density lipoprotein cholesterol (HDL-C) were analyzed using an automated chemistry analyzer (cobas^®^ c 702 module, Roche Diagnostics, Mannheim, Germany) with enzymatic colorimetric methods. Internal quality control procedures were performed daily; intra- and inter-assay coefficients of variation were maintained below 3–5%. Units were reported in mg/dL.

#### 2.2.3 Anthropometric and body composition assessment

Body weight and height were measured using a calibrated digital scale and stadiometer (Tanita, Japan). Body mass index (BMI) was calculated as weight in kilograms divided by height in meters squared (kg/m^2^). Waist circumference (WC) was measured at the midpoint between the lower margin of the last palpable rib and the top of the iliac crest, following the World Health Organization recommendations (29). Body composition, including appendicular skeletal muscle mass (ASM), was assessed using bioelectrical impedance analysis (InBody 770, InBody Co., Seoul, Korea), which has been validated in older populations. ASM was expressed both as absolute mass (kg) and relative to body weight (ASM%) (30).

#### 2.2.4 Muscle strength assessment

Grip strength was assessed using a digital hand dynamometer (T.K.K. 5,401, Takei Scientific Instruments Co., Ltd., Japan). The participants performed two maximal voluntary contractions for each hand in a standing position with the arms fully extended, and the highest value was recorded. Relative handgrip strength (RGS) was calculated as absolute handgrip strength (kg) divided by body weight (kg) and reported as kg/BW (31, 32).

Lower-limb muscle strength (LLMS) of the dominant leg was measured at an angular velocity of 60°/s using the HUMAC NORM isokinetic dynamometer (CSMi, USA) (49). The participants were seated with their hips and knees at 90° and asked to perform maximal voluntary knee extension. The highest peak torque across three trials was recorded, and relative LLMS was calculated as peak torque (Nm) divided by body weight (kg) and reported as Nm/BW.

#### 2.2.5 Cardiorespiratory fitness testing

Cardiorespiratory fitness (CRF) was evaluated using a graded treadmill exercise test following the Bruce protocol. During the test, expired gases were analyzed breath by breath using a metabolic cart (CASE8000, SensorMedics, USA). Before each testing session, the flow sensor was calibrated with a certified three-liter syringe, and the gas analyzers were calibrated with room air and a certified reference gas according to the manufacturer’s instructions. The highest oxygen consumption achieved during the test was recorded as VO₂max and expressed in mL·kg^−1^·min^-1^ (33).

#### 2.2.6 Metabolic syndrome diagnosis

MetS was defined according to the International Diabetes Federation (IDF) criteria, requiring the presence of central obesity (waist circumference ≥90 cm for male individuals and ≥80 cm for female individuals in Asian populations), plus at least two of the following additional factors: (1) Elevated triglycerides: ≥150 mg/dL (1.7 mmol/L), or specific treatment for this lipid abnormality; (2) reduced high-density lipoprotein cholesterol (HDL-C) levels: <40 mg/dL (1.03 mmol/L) in male individuals and <50 mg/dL (1.29 mmol/L) in female individuals, or receiving treatment for dyslipidemia; (3) elevated blood pressure (BP): systolic BP ≥ 130 mmHg or diastolic BP ≥ 85 mmHg, or on antihypertensive medication; and (4) elevated fasting plasma glucose: ≥100 mg/dL (5.6 mmol/L), previously diagnosed type 2 diabetes, or receiving antidiabetic treatment (34).

### 2.3 Statistical analysis

All analyses were performed using SPSS 25.0 (IBM). Continuous variables were reported as mean ± SD, and categorical variables as percentages. Group differences by MetS status were tested within each sex using *t*-tests (continuous) or χ^2^ tests (categorical). Sex-stratified logistic regression models were fitted with exposures standardized within sex, so that odds ratios represent the change in odds per one SD decrease for the following: relative grip strength (kg/BW), relative lower-limb strength (Nm/BW), VO₂max (mL·kg^−1^·min^−1^), and the ASM ratio (%). Two model sets were estimated: Model 1 adjusted for age and Model 2 additionally adjusted for BMI. Model calibration and discrimination were assessed using the Hosmer–Lemeshow test and the area under the receiver operating characteristic curve with 95% confidence intervals. Sex-specific receiver operating characteristic analyses were conducted to derive Youden cutoffs, and the sensitivity and specificity at the selected cutoffs are reported with 95% confidence intervals, obtained using a percentile bootstrap with 2,000 resamples. Multicollinearity was examined using variance inflation factors. Analyses were conducted using complete cases; a two-sided *p*-value of < 0.05 was considered statistically significant.

## 3 Results

### 3.1 Characteristics of the participants

A total of 570 middle-aged adults (369 male and 201 female) were included. The overall prevalence of MetS was 25.3% (95% CI 21.9–29.0); by sex, 25.5% in the male individuals (95% CI 21.3–30.2) and 24.9% in the female individuals (95% CI 19.4–31.3). Age did not differ by sex (*p* = 0.171). Compared to the female individuals, the male individuals were taller, heavier, and had higher BMI, WC, and ASM scores; they also showed higher DBP, TG, and fasting glucose levels, as well as greater GS, LLMS, and VO₂max (all *p* < 0.05). The female individuals had a higher body-fat percentage (*p* < 0.001). SBP and HDL-C did not differ significantly between sexes (*p* = 0.619 and 0.097, respectively) (Table 1).

**Table 1:** Characteristics of participants (mean ± SD).

Variables	Total (*N* = 570)	Men (*N* = 369)	Women (*N* = 201)	t	*P*-value
Age, years	54.2 ± 5.7	53.9 ± 5.1	54.6 ± 6.6	−1.370	0.171
Height, cm	165.2 ± 9.0	170.3 ± 6.0	155.9 ± 5.6	28.430	<0.001*
Weight, kg	66.3 ± 10.6	70.9 ± 8.9	57.9 ± 7.9	17.943	<0.001*
BMI, kg/m^2^	24.2 ± 2.7	24.4 ± 2.5	23.8 ± 3.1	2.405	0.017*
WC, cm	83.9 ± 8.3	85.9 ± 7.8	80.2 ± 8.0	8.206	<0.001*
Body fat percent, %	22.7 ± 6.4	19.6 ± 4.8	28.3 ± 5.0	−20.098	<0.001*
ASM, kg	15.1 ± 3.2	17.1 ± 1.9	11.6 ± 1.5	38.591	<0.001*
SBP, mmHg	121.9 ± 15.7	122.1 ± 14.9	121.4 ± 17.1	0.477	0.634
DBP, mmHg	76.6 ± 9.9	77.2 ± 10.1	75.3 ± 9.4	2.205	0.028*
TG, mg/dL	127.7 ± 74.3	133.7 ± 81.6	116.8 ± 57.2	2.886	0.004*
HDL-C, mg/dL	56.6 ± 14.4	55.9 ± 14.5	58.0 ± 14.2	−1.672	0.095
FG, mg/dL	102.7 ± 20.9	105.7 ± 24.0	97.2 ± 11.8	5.624	<0.001*
GS, kg	32.7 ± 9.3	38.3 ± 5.9	22.6 ± 4.6	35.114	<0.001*
LLMS, Nm	359.1 ± 119.3	423.8 ± 91.0	240.2 ± 57.3	29.484	<0.001*
VO₂max, mL·kg^−1^·min^−1^	29.9 ± 7.6	30.1 ± 7.9	29.5 ± 6.9	0.913	0.361

BMI, body mass index; WC, waist circumference; ASM, appendicular skeletal muscle; SBP, systolic blood pressure; DBP, diastolic blood pressure; TG, triglycerides; HDL-C, high-density lipoprotein cholesterol; FG, fasting glucose; GS, grip strength; LLMS, lower-limb muscle strength; VO₂max, maximal oxygen uptake. *p*-values and t-statistics were derived from independent samples *t*-tests comparing males and females.

### 3.2 Differences by MetS status

After stratifying by sex, the participants with MetS showed a consistently less favorable body-composition profile. In both male and female individuals, BMI, fat mass, and body-fat percentage were higher in the MetS group (all *p* < 0.001). Although absolute ASM was slightly greater in the MetS group, the ASM ratio (ASM/weight × 100) was lower in both sexes (male: *p* < 0.001; female: *p* < 0.001). Age was modestly higher in the participants with MetS (male: *p* = 0.016; female: *p* = 0.034). For muscle strength, absolute handgrip strength did not differ by MetS status (male: *p* = 0.420; female: *p* = 0.821), whereas relative grip strength (kg/BW) was lower in the MetS group for both sexes (both *p* < 0.001). Absolute lower-limb strength did not differ in the male individuals (*p* = 0.508) but was higher in the female individuals with MetS (*p* < 0.001); when normalized to body weight, relative lower-limb strength was lower in the male individuals with MetS (*p* < 0.001) and did not differ in the female individuals (*p* = 0.725). Cardiorespiratory fitness expressed as absolute VO₂ (L/min) did not differ (male: *p* = 0.660; female: *p* = 0.530), whereas relative VO₂max (mL·kg^−1^·min^−1^) was lower in the MetS group (male: *p* = 0.047; female: *p* = 0.007). Collectively, weight-normalized indicators (relative grip strength, relative lower-limb strength [in male individuals), and VO₂max (in both sexes)] discriminated MetS status better than absolute measures (Table 2).

**Table 2:** Body composition and fitness differences according to MetS status by sex (mean ± SD).

Variable	Men non-MetS	Men MetS	*P*	Women non-MetS	Women MetS	*P*
Age, years	53.5 ± 4.9	55.0 ± 5.5	0.016*	54.1 ± 7.0	56.4 ± 5.1	0.034*
BMI, kg/m^2^	23.7 ± 2.0	26.5 ± 2.6	<0.001*	22.9 ± 2.6	26.5 ± 2.7	<0.001*
Fat mass, kg	12.9 ± 3.6	18.0 ± 5.7	<0.001*	15.2 ± 4.3	21.0 ± 4.1	<0.001*
Body fat percent, %	18.5 ± 4.0	22.9 ± 5.4	<0.001*	27.0 ± 4.8	32.4 ± 3.3	<0.001*
ASM mass, kg	16.9 ± 1.8	17.7 ± 2.2	<0.001*	11.4 ± 1.5	12.1 ± 1.3	<0.001*
ASM ratio, %	24.6 ± 1.6	23.0 ± 1.9	<0.001*	20.5 ± 1.9	18.9 ± 1.1	<0.001*
GS, kg	38.4 ± 5.8	37.8 ± 6.1	0.420	22.6 ± 4.4	22.7 ± 5.2	0.821
GS, kg/BW	56.1 ± 7.8	49.9 ± 10.2	<0.001*	40.8 ± 8.1	35.3 ± 6.9	<0.001*
LLMS, Nm	425.7 ± 87.5	418.4 ± 100.9	0.508	231.6 ± 53.6	266.1 ± 60.9	<0.001*
LLMS, Nm/BW	6.2 ± 1.1	5.5 ± 1.3	<0.001*	4.2 ± 1.0	4.1 ± 0.8	0.725
VO₂, L/min	2.4 ± 0.7	2.4 ± 0.5	0.660	1.6 ± 0.4	1.5 ± 0.3	0.530
VO₂max, mL·kg^−1^·min^−1^	34.5 ± 9.1	31.6 ± 6.9	0.047*	27.7 ± 6.0	24.4 ± 6.1	0.007*

MetS, metabolic syndrome; BMI, body mass index; ASM, appendicular skeletal muscle; ASM ratio, ASM mass/body weight × 100; BW, body weight; GS, grip strength; LLMS, lower-limb muscle strength; VO₂/VO₂max, oxygen uptake (absolute/relative, respectively). *p*-values are from independent-samples *t*-tests within sex. * Indicates *p* < 0.05.

### 3.3 Associations with MetS

In sex-stratified logistic models, several weight-normalized indicators were associated with prevalent MetS before adjustment for BMI. Among the male individuals, a 1-SD decrease in relative grip strength (OR 2.05, 95% CI 1.56–2.68, *p* < 0.001), relative lower-limb strength (OR 1.92, 95% CI 1.44–2.55, *p* < 0.001), and ASM ratio (OR 2.78, 95% CI 2.06–3.76, *p* < 0.001) was associated with higher odds of MetS, whereas VO₂max was not significant (OR 1.12, 95% CI 0.87–1.45, *p* = 0.365). After additional adjustment for BMI (Model 2), these associations were attenuated to null (e.g., relative grip: OR 0.91, 95% CI 0.64–1.31, *p* = 0.618), with model discrimination remaining acceptable (AUC ≈ 0.82) (Table 3). Among the female individuals, lower relative grip strength (OR 1.92, 95% CI 1.33–2.77, *p* < 0.001) and lower ASM ratio (OR 3.05, 95% CI 1.89–4.93, *p* < 0.001) were associated with higher odds of MetS in age-adjusted models; VO₂max was borderline (OR 1.40, 95% CI 1.00–1.96, *p* = 0.051), and relative lower-limb strength was not significant (OR 0.89, 95% CI 0.61–1.28, *p* = 0.521). With additional adjustment for BMI, the associations for grip strength, VO₂max, and ASM ratio were no longer significant (all *p* > 0.40), while relative lower-limb strength showed an inverse association (OR 0.39, 95% CI 0.23–0.68, *p* = 0.001), indicating a suppression/over-adjustment effect in the fully adjusted model; overall discrimination remained good (AUC ≈ 0.84–0.85) (Table 4). Model discrimination for Model 2 was acceptable to good across exposures: in the male individuals, AUCs ranged from 0.815 to 0.818, with 95% CIs approximately 0.759–0.873; in the female individuals, AUCs ranged from 0.836 to 0.850, with 95% CIs approximately 0.761–0.920. Calibration (Hosmer–Lemeshow p) varied by exposure: male individuals: 0.000–0.015 and female individuals: 0.000–0.142. No important multicollinearity was detected (all VIF < 2.5). AUC values are presented with 95% confidence intervals; sensitivity and specificity at the Youden cutoffs are reported in Supplementary Table S1. In sensitivity analyses excluding BMI, associations per 1 SD decrease remained directionally consistent and were larger in magnitude; after including BMI, estimates were attenuated and several confidence intervals widened (Supplementary Table S2).

**Table 3:** Multivariable logistic regression for MetS (Men).

Exposure (per 1-SD decrease)	n	Model 1 OR (95% CI) [Adjusted for age]	*P*	Model 2 OR (95% CI) [+ BMI]	P	AUC (Model 2)
Relative grip strength	369	2.05 (1.56, 2.68)	0.000	0.91 (0.64, 1.31)	0.618	0.815
Relative leg strength	369	1.92 (1.44, 2.55)	0.000	1.24 (0.88, 1.73)	0.215	0.815
VO₂max	369	1.12 (0.87, 1.45)	0.365	1.13 (0.83, 1.55)	0.434	0.818
ASM ratio	369	2.78 (2.06, 3.76)	0.000	1.04 (0.68, 1.59)	0.851	0.815

MetS, metabolic syndrome; OR, odds ratio; CI, confidence interval; AUC, area under the ROC curve; BMI, body mass index; ASM, appendicular skeletal muscle; BW, body weight; VO₂max, maximal oxygen uptake. ORs are per 1-SD decrease in exposure. Model 1 adjusted for age; Model 2 additionally adjusted for BMI. AUC refers to Model 2. Two-sided *p* < 0.05.

**Table 4:** Multivariable logistic regression for MetS (Women).

Exposure (per 1-SD decrease)	n	Model 1 OR (95% CI) [Adjusted for age]	P	Model 2 OR (95% CI) [+ BMI]	P	AUC (Model 2)
Relative grip strength	201	1.92 (1.33, 2.77)	0.000	1.02 (0.64, 1.63)	0.935	0.842
Relative leg strength	201	0.89 (0.61, 1.28)	0.521	0.39 (0.23, 0.68)	0.001	0.850
VO₂max	197	1.40 (1.00, 1.96)	0.051	1.08 (0.76, 1.55)	0.659	0.836
ASM ratio	201	3.05 (1.89, 4.93)	0.000	1.29 (0.70, 2.36)	0.412	0.845

MetS, metabolic syndrome; OR, odds ratio; CI, confidence interval; AUC, area under the ROC curve; BMI, body mass index; ASM, appendicular skeletal muscle; BW, body weight; VO₂max, maximal oxygen uptake. ORs are per 1-SD decrease in exposure. Model 1 adjusted for age; Model 2 additionally adjusted for BMI. AUC refers to Model 2. Two-sided *p* < 0.05.

### 3.4 Discrimination and cutoffs

**Figure 1:**
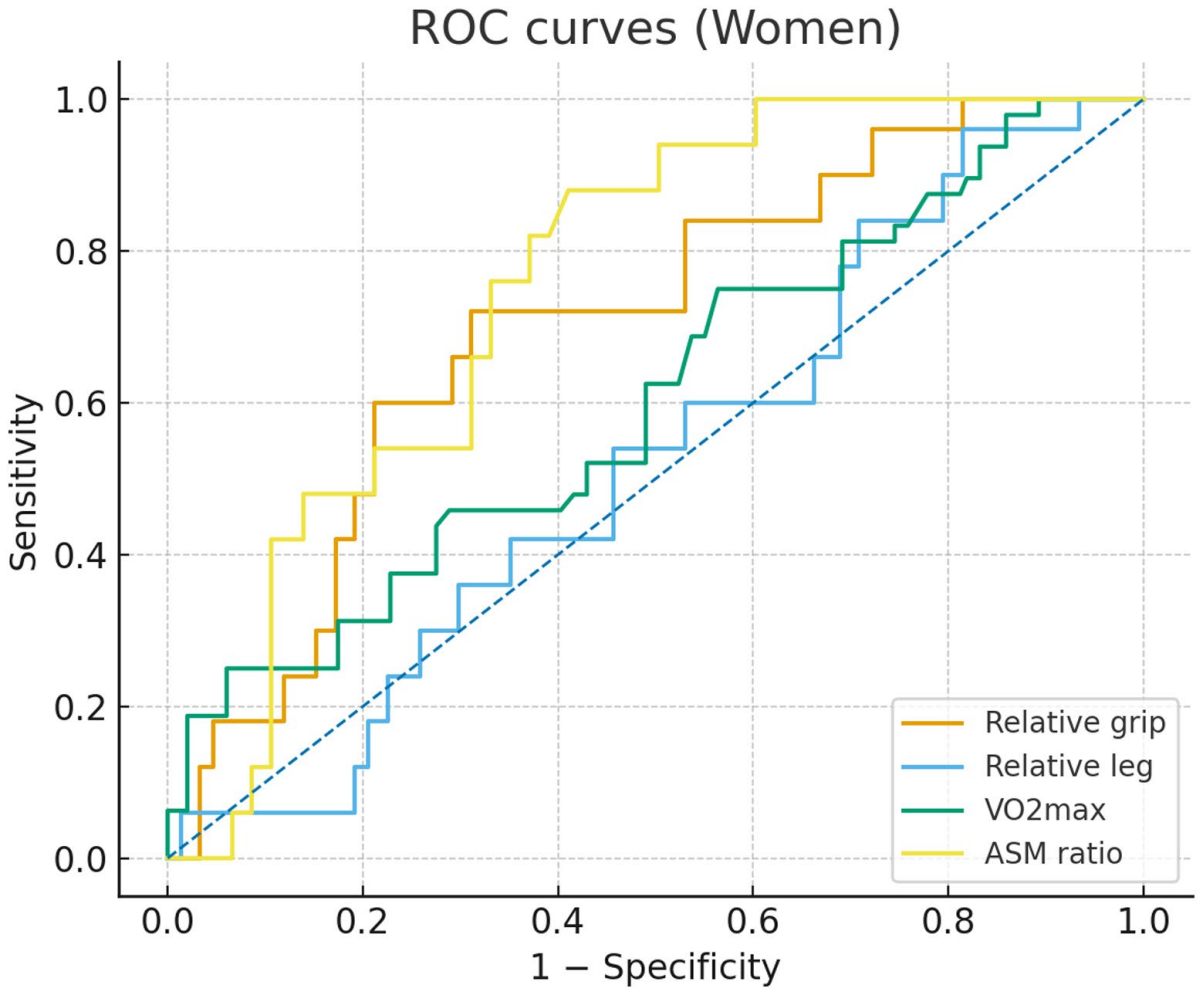
Receiver operating characteristic (ROC) curves for women comparing relative grip strength (orange), relative lower-limb strength (blue), VO2max (green), and appendicular skeletal muscle (ASM) ratio (yellow). Sensitivity is plotted against 1 - specificity.

**Figure 2:**
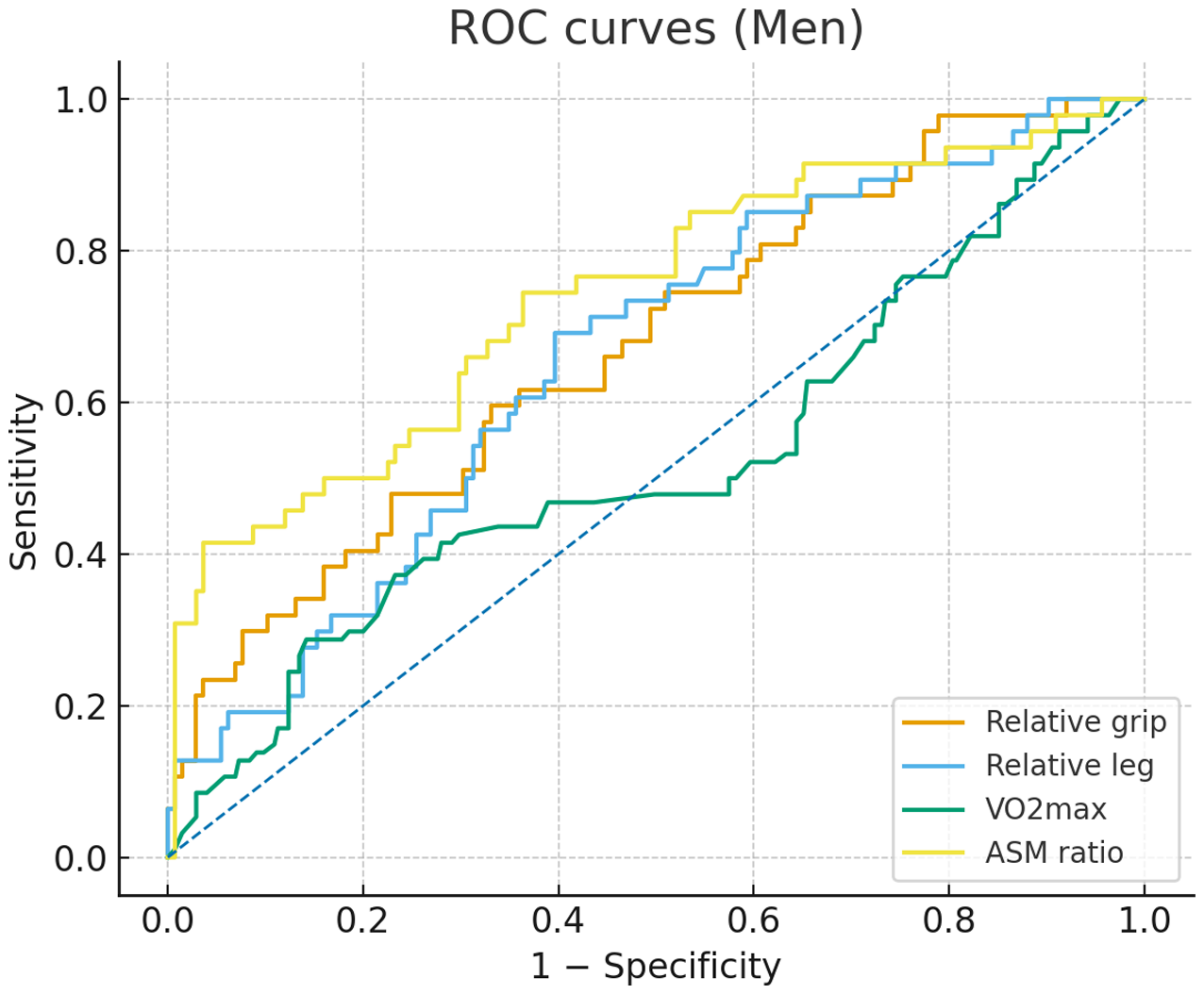
Receiver operating characteristic (ROC) curves for men comparing relative grip strength (orange), relative lower-limb strength (blue), VO2max (green), and appendicular skeletal muscle (ASM) ratio (yellow). Sensitivity is plotted against 1 - specificity.

In sex-specific ROC analyses (Figures 1, 2), the ASM ratio showed the best discrimination of MetS in both sexes—the ROC curve lay farthest from the diagonal reference. In the male individuals, AUCs were 0.74 for the ASM ratio, 0.67 for relative grip strength, 0.66 for relative leg strength, and 0.52 for VO₂max, indicating limited discrimination for VO₂max. In the female individuals, AUCs were 0.75 for the ASM ratio, 0.70 for relative grip strength, 0.60 for VO₂max, and 0.52 for relative leg strength, the latter approximating chance. The Youden index cutoffs and exact 95% CIs of the AUCs are provided in Supplementary Table S1; ROC curves are shown in Figures 1, 2.

## 4 Discussion

In this sex-stratified cross-sectional study of 570 middle-aged adults, weight-normalized indices showed the clearest associations with metabolic syndrome. Among the four domains, the ASM ratio provided the best discrimination in both sexes, followed by RGS. Relative lower-limb strength discriminated modestly in the male individuals and approached chance in the female individuals, whereas VO₂max contributed little in the male individuals and only modestly in the female individuals. In logistic models scaled per 1 SD decrease in exposure, associations were evident after adjusting for age and were attenuated after additional adjustment for BMI, which is compatible with partial overadjustment through adiposity. In the female individuals, the coefficient for relative lower-limb strength shifted toward the null and became positive in the BMI-adjusted model, indicating a suppression effect related to collinearity between body size and strength. Taken together, weight-normalized measures, especially the ASM ratio and relative grip strength, captured metabolic risk more effectively than absolute metrics and support the use of sex-specific cutoffs for risk stratification in middle-aged adults. These results should be viewed as complementary to integrated phenotyping rather than as competing alternatives. Our thresholds provide feasible screening anchors that can be combined with waist circumference or body fat measures to enhance discrimination and reclassification, in line with prior research that coupled central adiposity with relative strength and composite scores that include body fat percentage, waist circumference, and grip strength (23, 25). Framing our findings within the cardiometabolic-based chronic disease model and a functional body composition perspective clarifies why weight-normalized functional indices capture risk beyond BMI and highlights the need for prospective validation of combined models in Chinese and other Asian populations (26, 27).

Our findings extend the literature linking muscular fitness with metabolic risk and clarify that weight-normalized metrics are more informative than absolute values in middle-aged populations. Large analyses and cohort studies have reported inverse associations between handgrip strength and insulin resistance, metabolic syndrome, and cardiovascular events, particularly when strength is scaled to body size rather than analyzed in absolute kilograms (13, 16, 17). Consistent with this evidence, RGS outperformed absolute grip for discriminating metabolic syndrome in our cohort (35). This aligns with the concept that muscle function relative to the mechanical load of body mass is more tightly linked to metabolic health than absolute force production. Since heavier individuals can show high absolute grip strength despite adverse adiposity profiles, normalization by body weight reduces this masking and improves the interpretability of strength as a health marker (13, 16). Regarding muscle mass, previous research comparing absolute appendicular skeletal muscle with body size-adjusted metrics suggests that muscle proportion is more relevant than quantity alone for metabolic profiling (10). Our observation that the ASM ratio achieved the highest discrimination in both sexes supports this view and indicates that the balance between lean mass and body mass carries more information than raw lean mass (36). Absolute ASM is strongly correlated with body size, which can obscure clinically meaningful gradients in risk when not scaled. The ASM ratio, by contrast, reflects the relative capacity of the musculature to support glucose disposal and lipid oxidation at a given body mass, which is a more direct determinant of metabolic efficiency. Evidence on isokinetic lower-limb strength is more limited than for handgrip. The classic literature established functionally meaningful thresholds for quadriceps torque, but it did not focus on metabolic clustering, and absolute knee extensor torque is strongly influenced by body mass and fat mass (19). This influence can inflate absolute values without reflecting functional adequacy relative to body size. Our results fit this pattern. Once lower-limb strength was expressed relative to body weight and BMI was included in the model, discrimination was modest in the male individuals and near chance in the female individuals. The sex difference may reflect differences in fat distribution, segmental mass, and habitual activity patterns, which alter the coupling between torque production and metabolic traits (37). Female individuals typically accumulate more iliofemoral and intramuscular fat with age, particularly around the menopausal transition, which can degrade muscle quality without large losses in absolute torque, thereby weakening the relation between leg strength and metabolic risk after adiposity adjustment. For cardiorespiratory fitness, prospective cohorts often report stronger relations with incident cardiometabolic outcomes than with cross-sectional metabolic syndrome (21, 22). Our data follow this pattern, with VO₂max showing little value in the male individuals and modest value in the female individuals (38). As shown in Supplementary Table S2, estimates were larger without BMI and were attenuated after adding BMI. Several factors may explain this result. Midlife cohorts often show narrower variability in measured or estimated VO₂max than in adiposity or muscular traits. Field testing and submaximal estimation introduce measurement errors that can attenuate associations. The clustering of metabolic syndrome components at midlife is driven strongly by adiposity and insulin resistance, which may weaken the cross-sectional contribution of aerobic capacity in models that already include body size-related variables (21, 22). Our findings underscore the importance of declaring the role of adiposity when interpreting associations for weight-normalized exposures. For these metrics, BMI is not a simple confounder. It is closely related to the denominator and partly mediates the pathway from muscular fitness to metabolic risk. Therefore, treating BMI as a secondary adjustment avoids overinterpretation of ‘independent’ effects and provides a transparent view of how adiposity influences the apparent strength of association. To place these statistical patterns in a physiological context, we briefly outline the measurement rationale and underlying biology.

Mechanistically, absolute strength and oxygen uptake rise with body size, so heavier individuals can appear stronger or fitter in absolute terms despite higher metabolic risk. Normalizing by body weight yields indices that better reflect functional capacity relative to metabolic load. Skeletal muscle is the principal site for insulin-stimulated glucose disposal, and a smaller muscle compartment relative to body size can amplify insulin resistance, elevate fasting glucose, and worsen lipid handling (6). Muscle quality also matters. Greater intramuscular fat and lower mitochondrial efficiency reduce strength per unit muscle and promote proinflammatory signaling, which further impairs insulin sensitivity (39). Relative grip strength likely captures these quality features because it reflects neuromuscular function in a task that is minimally confounded by whole-body support, while the ASM ratio captures the structural capacity of the lean compartment relative to total mass. A lower muscle-to-fat ratio is plausibly linked to impaired metabolic control through several converging pathways. Greater intramuscular adipose infiltration elevates lipid intermediates such as diacylglycerols and ceramides, activates protein kinase C isoforms, and reduces insulin receptor substrate phosphorylation, which together blunt downstream PI3K/Akt signaling and limit GLUT4 translocation in skeletal muscle (40, 41). Concomitant reductions in mitochondrial oxidative capacity and biogenesis constrain fatty acid oxidation and increase reactive oxygen species, a milieu that favors ectopic lipid deposition and further insulin resistance (42, 43). The muscle secretome may contribute as well. Lower muscle mass and reduced contractile activity are associated with a myokine profile characterized by higher myostatin and lower levels of exercise-induced, metabolically favorable myokines, such as IL-6 in its transient form, irisin, and IL-15. These shifts can dampen adipose tissue browning, decrease whole-body energy expenditure, and promote low-grade inflammation (44, 45). Through these mechanisms, a low muscle-to-fat ratio can plausibly worsen fasting glucose, elevate triglycerides, and lower HDL-cholesterol, and it may also influence blood pressure via endothelial dysfunction and sympathetic activation. While our study was not designed to adjudicate these pathways, the observed associations align with this mechanistic framework and provide biological credibility to the statistical results.

These findings have immediate applicability in routine health checks for middle-aged adults. Two simple and scalable indices, the ASM ratio and relative grip strength, provided the most effective discrimination of metabolic syndrome and support sex-specific cutoffs derived from our ROC analyses. Values below these thresholds can signal higher metabolic risk during a standard visit and justify targeted counseling and laboratory evaluation. In practice, these measures complement BMI and waist circumference. When adiposity measures are borderline or discordant, the addition of relative grip strength and the ASM ratio can refine risk stratification in a way that is interpretable at the point of care, because lower values directly indicate an unfavorable balance between muscle and body mass. Comparative relevance to composite indicators: prior research shows that the waist-to-height ratio, muscle-to-fat composites, and continuous metabolic syndrome severity scores discriminate cardiometabolic risk (23, 27, 46, 47). Our findings indicate that the ASM ratio and relative grip strength capture a muscular domain not fully represented by these adiposity-dominant or multi-component indices; therefore, they provide complementary information for screening. Formal tests of incremental value will be addressed in future prespecified models.

The study has notable strengths, including standardized field measurements across multiple fitness domains, sex-stratified modeling, and clinically interpretable thresholds derived from ROC analysis. However, there are limitations. First, the cross-sectional design precludes causal inference. Second, the cohort consisted of middle-aged Korean adults from a single center, which limits generalizability, and the ROC-derived thresholds were not internally validated, highlighting the need for external validation in more diverse populations. Third, because exposures were referenced to body weight, additional adjustment for BMI may induce overadjustment. Fourth, we did not benchmark the ASM ratio and relative grip strength against composite indicators or quantify their incremental value, which should be addressed in future studies. Finally, despite these limitations, the consistent patterns across analyses support the practical utility of weight-referenced indices, particularly the ASM ratio and relative grip strength, for screening in this age group.

## 5 Conclusion

In this sex-stratified cross-sectional study of 570 middle-aged adults, weight-normalized measures best captured metabolic syndrome risk. The ASM ratio and relative grip strength showed the strongest discrimination; relative lower-limb strength was informative in the male individuals but not in the female individuals, and VO₂max contributed little in the male individuals and only modestly in the female individuals. The derived sex-specific cutoffs for the ASM ratio and relative grip strength can support screening and risk stratification in clinical and community checkups, with the understanding that they are not diagnostic.

## Data Availability

The original contributions presented in the study are included in the article/Supplementary material, further inquiries can be directed to the corresponding author.
