# Supplementary material for: Associations between physical fitness components and metabolic syndrome in middle-aged adults: a cross-sectional study using relative strength indicators and ROC analysis
Source: Front Public Health. 2025 Nov 17;13:1712376. doi: 10.3389/fpubh.2025.1712376 (PMC12665587; doi:10.3389/fpubh.2025.1712376)
Supplement: Supplementary file 1 [file Table_1.DOCX]

**Supplementary Table S1. ROC metrics by exposure (Men)**

| **Exposure** | **n** | **AUC (95% CI)** | **Cut-off (Unit)** | **Sensitivity** | **Specificity** |
| --- | --- | --- | --- | --- | --- |
| Relative grip strength | 369 | 0.67 (0.61–0.74) | 53.33 (kg/BW) | 0.60 | 0.67 |
| Relative leg strength | 369 | 0.66 (0.59–0.72) | 5.94 (Nm/BW) | 0.69 | 0.60 |
| VO2max | 369 | 0.52 (0.46–0.59) | 24.00 (mL/kg/min) | 0.29 | 0.86 |
| ASM ratio | 369 | 0.74 (0.68–0.80) | 24.02 (%) | 0.74 | 0.64 |

**Supplementary Table S1. ROC metrics by exposure (Women)**

| **Exposure** | **n** | **AUC (95% CI)** | **Cut-off (Unit)** | **Sensitivity** | **Specificity** |
| --- | --- | --- | --- | --- | --- |
| Relative grip strength | 201 | 0.70 (0.61–0.79) | 37.98 (kg/BW) | 0.72 | 0.69 |
| Relative leg strength | 201 | 0.52 (0.43–0.62) | 5.03 (Nm/BW) | 0.96 | 0.19 |
| VO2max | 197 | 0.60 (0.51–0.70) | 19.30 (mL/kg/min) | 0.25 | 0.94 |
| ASM ratio | 201 | 0.75 (0.67–0.84) | 20.00 (%) | 0.88 | 0.59 |

Notes: AUC 95% CIs were calculated using the Hanley–McNeil method. Cut-offs were selected by the Youden index; lower exposure indicates higher risk.
