# Supplementary material for: Associations between physical fitness components and metabolic syndrome in middle-aged adults: a cross-sectional study using relative strength indicators and ROC analysis
Source: Front Public Health. 2025 Nov 17;13:1712376. doi: 10.3389/fpubh.2025.1712376 (PMC12665587; doi:10.3389/fpubh.2025.1712376)
Supplement: Supplementary file 2 [file Table_2.DOCX]

**Supplementary Table S2. BMI sensitivity analysis: models without and with BMI**

| **Exposure** | **Men Model 1 OR**  **(95% CI), p** | **Men Model 2 OR**  **(95% CI), p** | **Women Model 1 OR**  **(95% CI), p** | **Women Model 2 OR**  **(95% CI), p** | **AUC**  **(Model 2) Men** | **AUC (Model 2) Women** |
| --- | --- | --- | --- | --- | --- | --- |
| Relative grip strength (kg/BW) | 2.05 (1.56–2.68), p<0.001 | 0.91 (0.64–1.31), p=0.618 | 1.92 (1.33–2.77), p<0.001 | 1.02 (0.64–1.63), p=0.935 | 0.815 | 0.842 |
| Relative leg strength (Nm/BW) | 1.92 (1.44–2.55), p<0.001 | 1.24 (0.88–1.73), p=0.215 | 0.89 (0.61–1.28), p=0.521 | 0.39 (0.23–0.68), p=0.001 | 0.815 | 0.850 |
| VO₂max (mL·kg⁻¹·min⁻¹) | 1.12 (0.87–1.45), p=0.365 | 1.13 (0.83–1.55), p=0.434 | 1.40 (1.00–1.96), p=0.051 | 1.08 (0.76–1.55), p=0.659 | 0.818 | 0.836 |
| ASM ratio (%) | 2.78 (2.06–3.76), p<0.001 | 1.04 (0.68–1.59), p=0.851 | 3.05 (1.89–4.93), p<0.001 | 1.29 (0.70–2.36), p=0.412 | 0.815 | 0.845 |

Notes: Values are transcribed from main Tables 3, 4. This table presents the two specifications side by side to illustrate attenuation attributable to BMI. Exposures: relative grip (kg/BW), relative leg (Nm/BW), VO₂max (mL·kg⁻¹·min⁻¹), ASM ratio (%).
